# Reliability of the balance evaluation systems test and trunk control measurement scale in adult spinal deformity

**DOI:** 10.1371/journal.pone.0221489

**Published:** 2019-08-26

**Authors:** Pieter Severijns, Thomas Overbergh, Lennart Scheys, Lieven Moke, Kaat Desloovere

**Affiliations:** 1 Institute for Orthopaedic Research and Training (IORT), Department of Development and Regeneration, Faculty of Medicine, KU Leuven, Leuven, Belgium; 2 Department of Rehabilitation Sciences, KU Leuven, Leuven, Belgium; 3 Clinical Motion Analysis Laboratory (CMAL), University Hospitals Leuven, Leuven, Belgium; 4 Division of Orthopaedics, University Hospitals Leuven, Leuven, Belgium; Toronto Rehabilitation Institute - UHN, CANADA

## Abstract

**Objective:**

To test the reliability of the Balance Evaluation Systems Test (BESTest) and Trunk Control Measurement Scale (TCMS) between sessions and raters in the adult spinal deformity (ASD) population.

**Summary of background data:**

Up to now evaluation in ASD was mainly based on static radiographic parameters. Recently literature showed that dynamic balance was a better predictor of health-related quality of life than radiographic parameters, stressing the importance of balance assessment. However, to the best of our knowledge, reliability of balance assessment tools has not yet been investigated in the ASD population.

**Methods:**

Twenty ASD patients participated in this study. Ten patients were included in the test-retest study, including repeated measurements. Ten patients were measured once, simultaneously but independently by three raters. Each participant performed two balance scales, namely the BESTest and the TCMS. Statistical analysis consisted of intra class correlations (ICC) on scale- and subscale level, and kappa scores on item-level. Cronbach’s alpha on total scores, standard errors of measurement (SEM), smallest detectable differences and percentages of agreement were also calculated. Bland-altman plots were created to investigate systematic bias.

**Results:**

ICC scores between sessions and raters for TCMS (0.76 and 0.88) and BESTest (0.90 and 0.94) total scores were good to excellent. SEM’s between sessions and raters were also low for total scores on TCMS (1.66 and 2.35) and BESTest (2.99 and 2.32). However, on subscale- and item-level reliability decreased and ceiling effects were observed. No systematic bias was observed between sessions and raters.

**Conclusion:**

BESTest and TCMS showed to be reliable tools to measure balance in ASD on scale-level. However, on subscale- and item-level reliability decreased and ceiling effects were observed. Therefore, the question arises if there is need for an ASD-specific balance scale.

## Introduction

Adult spinal deformity (ASD) comprises a wide range of three-dimensional (3D) spinal malalignments [1], affecting both skeletal and soft-tissue structures [2]. Postural balance is the ability of the human body to maintain its center of mass (COM) within the base of support (BOS) with minimal postural sway, requiring coordination between both musculoskeletal and sensorineural systems [3]. Past literature already confirmed a relation between the presence of a spinal deformity and impaired postural balance [4,5], with a large amount of research specifically reporting on the role of spinal alignment in keeping the COM within the BOS during stance, and the compensation strategies used to correct for spinal malalignment [6–11]. However, evaluation of spinal alignment and compensation strategies is currently mainly based on radiographic parameters derived from static two-dimensional (2D) X-rays [12,13]. Due to the static character of this evaluation method, it fails to assess the dynamic properties of balance; inherently a dynamic concept, preserved, achieved and restored by postural control strategies [14].

A recent study confirmed the assumption that dynamic balance is compromised in ASD patients, by applying the Balance Evaluation Systems Test (BESTest) [15] and Trunk Control Measurement Scale (TCMS) [16] on a cohort of ASD patients and healthy controls [4]. Furthermore, the results of this study showed that performance on BESTest had higher potential to predict health-related quality of life (HRQOL) in ASD patients than demographic and 2D radiographic spinopelvic parameters [4], which form the current cornerstone of clinical decision-making within ASD. Also, other researchers reported that the relation between 2D radiographic spinopelvic parameters and HRQOL scores is weaker than previously assumed, questioning the current classification and evaluation methods in ASD [4,17,18].

Dynamic balance control thus seems to be related to 3D spinal alignment in ASD patients. In addition, dynamic balance control seems to be stronger related to HRQOL than 2D spinopelvic alignment. These previously reported relations, in combination with the static character of the current 2D evaluation methods, stress the need for reliable and valid balance assessment tools in ASD. Previous work showed that the BESTest and TCMS are able to discriminate between ASD patients and healthy controls on balance capacity [4]. The fact that the BESTest assesses different aspects of balance, combined with its possibility to identify balance disorders within different balance systems [15], makes the BESTest an interesting tool to use in ASD. The TCMS is developed to measure static and dynamic aspects of trunk control in the CP population [16]. Since the test is administered in a seated position, decreasing the possibility for lower limb compensation, the test is very useful to investigate trunk control during movement in the ASD population. Since the use of TCMS and BESTest for balance assessment within the ASD population has only been introduced recently, the aim of this paper is to evaluate the reliability of these two balance scales in the ASD population, both between sessions and raters, to further explore their potential for clinical use in ASD.

## Methods

### Ethics statement

This study was approved by the ethical committee of the university hospitals Leuven (S58082) and all subjects provided written informed consent.

### Participants

Twenty ASD patients were recruited from our outpatient spinal clinic, of which ten were included in the test-retest study and ten in the interrater study. Demographic parameters and 2D spinopelvic alignment analysis according to the SRS-Schwab classification [13] can be found in Table 1. Following inclusion criteria were applied: mini mental state examination ≥ 25; able to walk 50 meters independently; no current history of musculoskeletal disorders of the lower extremities; no history of neurological disease; no history of spinal fusion surgery.

**Table 1:** Sample size and demographic parameters.

	Test-retest (N = 10)	Interrater (N = 10)
**Demographic parameters**		
Age, yr	63.0 ± 13.3	59.6 ± 8.3
Body height, cm	166.4 ± 0.1	160.7 ± 0.1
Body weight, kg	65.3 ± 15.8	67.1 ± 5.9
Body mass index, kg/m^2^	23.3 ± 3.2	26.2 ± 3.3
Gender (M/F)	3/7	3/7
**2D Spinopelvic parameters**		
Pelvic tilt (°)	23.0 ± 10.9	20.4 ± 12.3
Sagittal vertical axis (mm)	40.2 ± 37.7	46.3 ± 67.7
Pelvic incidence minus lumbar lordosis (°)	8.8 ± 17.7	14.3 ± 34.9
SRS-Schwab Coronal classification (D/T/L/N)	7D/3L	4D/6L

D: Double; T: Thoracic; L: Lumbar; N: No coronal curve

### Testing procedure

To examine the test-retest reliability of the BESTest and TCMS, ten subjects were scored twice by one single trained physiotherapist on two different occasions. The interval between the two tests was maximum two weeks, to avoid changes in clinical status or progression of deformity. To avoid a learning effect, test and retest were never administered on the same day. To examine interrater reliability, ten participants were, simultaneously but independently, scored by an experienced physiotherapist and two trained master students physiotherapy. Test instructions were always provided by the physiotherapist, while all three raters scored the performance of the subject while being blinded to the scoring of each other.

The TCMS was always administered first followed by a resting period of 5 minutes. Before, between and after the balance assessments, pain was scored through the Visual Analog Scale (VAS) [19] to investigate if pain levels remained constant. VAS scores were analyzed between test and retest to assure clinical status had not changed.

### Balance assessment tools

The TCMS is a seated balance test that measures trunk control both statically and dynamically [16]. The test is divided into 3 subscales, namely ‘static sitting balance’, ‘selective movement control’ and ‘dynamic reaching’, and consists of 15 items. All items are scored on a two-, three- or four-point ordinal scale and administered bilaterally in case of clinical relevance. The total score of the TCMS ranges from 0 to 58, with a higher score indicating a better performance. The patient is sitting upright without back, arm or feet support to avoid upper or lower limb compensations. The test takes approximately 15 minutes to conduct in ASD patients [4].

The BESTest is a functionality scale developed to assess balance and risk of falls in the elderly [15]. It consists of 36 items and is grouped into six subsections, which represent different systems that may constrain balance, namely ‘biomechanical constraints’, ‘stability limits/verticality’, ‘anticipatory postural adjustments’, ‘postural responses’, ‘sensory orientation’, and ‘stability in gait’. Each item is scored on a four-point ordinal scale from 0 (worst performance) to 3 (best performance). Total and subscale scores are translated to a percentage score. The BESTest takes approximately 25 minutes to conduct in ASD patients [4].

### Statistical analysis

On scale- and subscale-level interrater and test-retest reliability were determined by intraclass correlation coefficients (ICC) with a two-way random effects model for absolute agreement for single measurement (ICC (2,1)). ICC values <0.50 were considered poor; from 0.50–0.75 moderate; from 0.75–0.90 good; and >0.90 excellent[20]. The standard error of measurement (SEM) and smallest detectable difference (SDD) were calculated according to the following formulas: SEM = SD x √(1-ICC); SDD = SEM x 1.96√2.[20] The internal consistency of the TCMS, BESTest and its subscales was calculated with Cronbach’s alpha. Values from 0.70–0.90 indicate strong internal consistency[20]. On item-level, Cohen’s kappa was calculated to investigate test-retest reliability, while a free marginal kappa [21], which is a kappa extension for multiple raters, was used to assess interrater reliability. Kappa values <0.41 were considered poor, from 0.41–0.60 moderate, from 0.61–0.80 good and >0.81 excellent [22]. Percentages of agreement between sessions and raters were reported. For both ICC and kappa scores 95% confidence intervals (CI) were calculated. The method by Bland and Altman was used to determine systematic bias between sessions and raters [23]. The level of significance was set at p<0.05. SPSS 24 was used for statistical analyses, except for free marginal kappa for which the online kappa calculator of Randolph et al. [21] was used.

## Results

### Scale- and subscale-level reliability

Test-retest and interrater reliability of the TCMS total score was good, with ICC’s of, respectively, 0.76 and 0.88. Reliability of the subscales of the TCMS was good to excellent, except for the test-retest reliability of ‘dynamic reaching’ (ICC = 0.27) which was found to be poor, and the interrater reliability of ‘static sitting balance’ (ICC = 0.65) which was found to be moderate. Test-retest and interrater reliability of the BESTest total score was found to be excellent with ICC’s of respectively 0.90 and 0.94. Reliability of the subscales of the BESTest was good to excellent, except for the test-retest reliability of ‘stability limits/verticality’ (ICC = 0.43) and ‘reactive postural response’ (ICC = 0.31) which were found to be poor, and the interrater reliability of ‘sensory orientation’ (ICC = 0.60) which was found to be moderate. The SEM’s between sessions and raters were low for total scores on TCMS (1.66 and 2.35) and BESTest (2.99 and 2.32). However, on subscale-level SEM’s were higher. Exact values for ICC, 95% confidence intervals, SEM and SDD for total and subscale scores are displayed in Table 2.

**Table 2:** Measures for test-retest and interrater reliability and internal consistency of BESTest and TCMS.

	Internal consistency	Test-retest (N = 10)					Interrater (N = 10)				
	Cronbach's α	ICC	Sig.	95% CI	SEM	SDD	ICC	Sig.	95% CI	SEM	SDD
**Total TCMS (0–58)**	0.70	0.88	**<0.001**	0.51–0.97	1.66	4.61	0.76	**<0.001**	0.44–0.93	2.35	6.52
Static sitting balance (0–20)	0.77	0.79	**0.002**	0.38–0.94	0.51	1.41	0.65	**<0.001**	0.31–0.89	0.66	1.82
Selective movement control (0–28)	0.71	0.86	**<0.001**	0.51–0.96	1.42	3.94	0.77	**<0.001**	0.42–0.93	1.82	5.05
Dynamic reaching (0–10)	0.79	0.27	0.223	-0.45–0.76	1.45	4.03	0.92	**<0.001**	0.80–0.98	0.48	1.33
**Total BESTest (%)**	0.70	0.90	**<0.001**	0.66–0.98	2.99	8.30	0.94	**<0.001**	0.83–0.98	2.32	6.43
Biomechanical constraints	0.50	0.88	**<0.001**	0.60–0.97	5.50	15.26	0.86	**<0.001**	0.65–0.96	6.33	17.55
Stability limits/verticality	0.63	0.43	0.109	-0.30–0.82	5.91	16.39	0.84	**<0.001**	0.61–0.95	3.24	8.97
Anticipatory postural adjustments	0.30	0.85	**<0.001**	0.51–0.96	6.37	17.66	0.92	**<0.001**	0.80–0.98	4.40	12.18
Reactive postural response	0.51	0.31	0.188	-0.39–0.77	12.39	34.33	0.85	**<0.001**	0.62–0.96	5.43	15.05
Sensory orientation	0.60	0.68	**0.013**	0.13–0.91	5.23	14.49	0.60	**0.001**	0.24–0.86	5.17	14.34
Stability in gait	0.36	0.81	**0.001**	0.41–0.95	5.27	14.61	0.91	**<0.001**	0.77–0.97	3.27	9.06

BESTest: Balance evaluation systems test; TCMS: Trunk control measurement scale; CI: Confidence interval; SEM: Standard error of measurement; SDD: Smallest detectable difference; Sig <0.05

Cronbach’s alpha of the total scores on both TCMS and BESTest was 0.70, indicating strong internal consistency. Cronbach’s alpha for each of the subscales can be found in Table 2.

**Fig 1:**
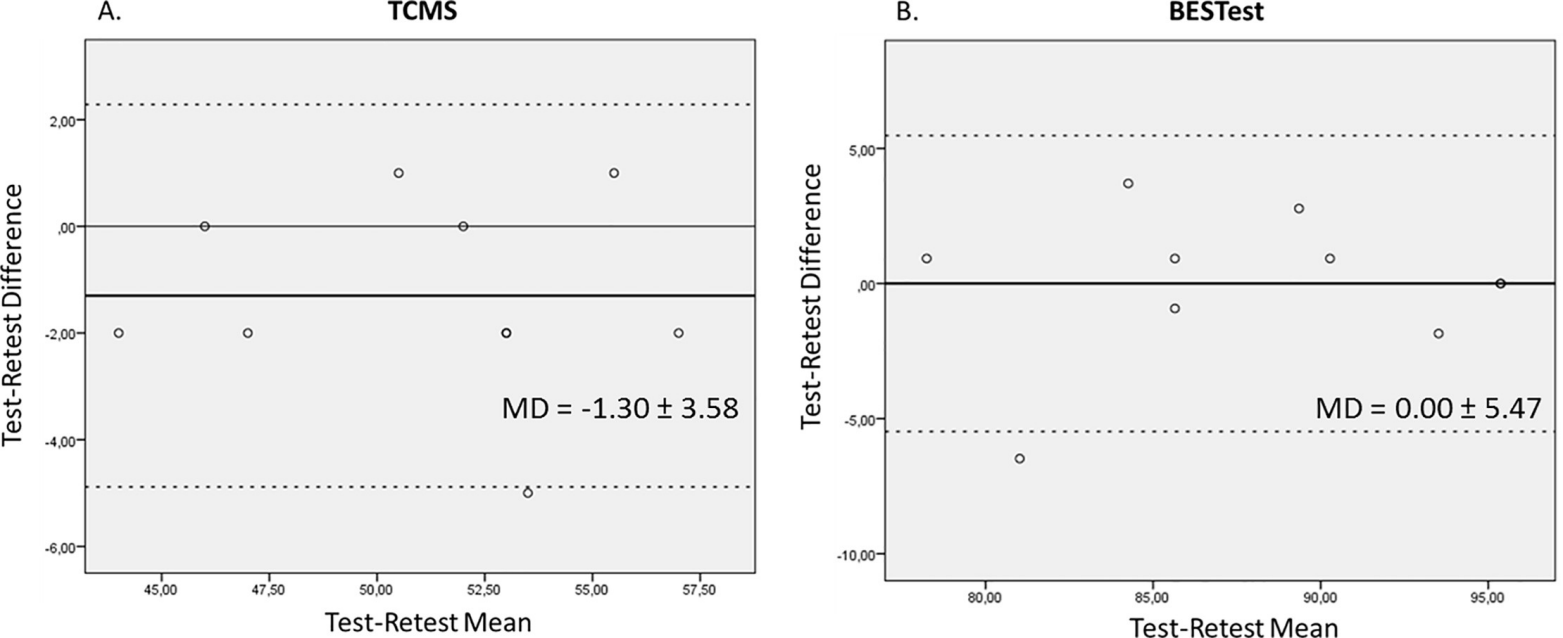
Bland-Altman plots for test-retest reliability of TCMS and BESTest. On the horizontal axis the mean score on TCMS and BESTest of the test and retest session is displayed. On the vertical axis the difference between test and retest is plotted. The solid thin line represents the zero axis. The bold black line represents the mean difference. The dotted lines indicate the limits of agreement. MD: Mean difference.

**Fig 2:**
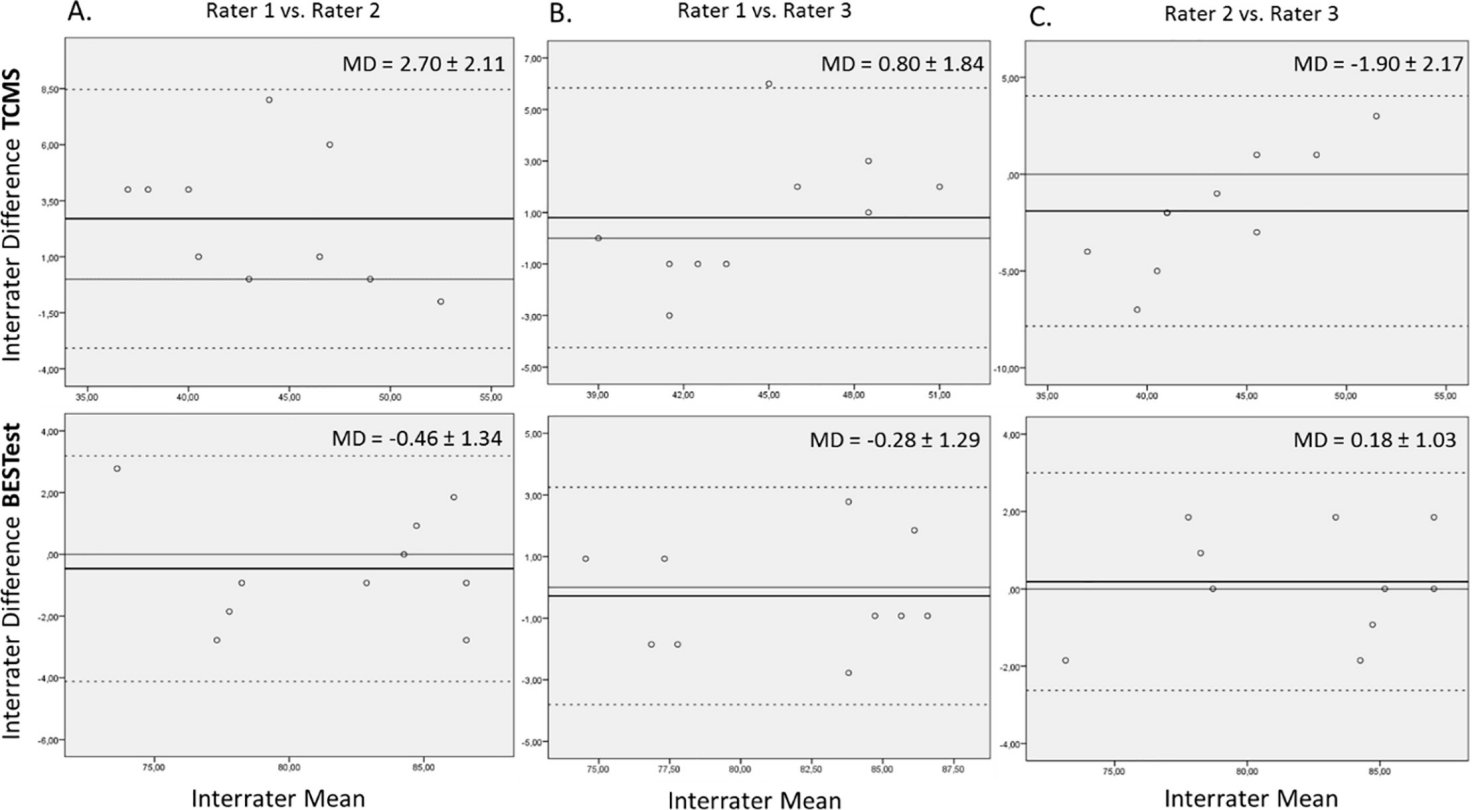
Bland-Altman plots for interrater reliability of TCMS and BESTest. On the horizontal axis the interrater mean score on TCMS and BESTest is displayed. On the vertical axis the difference between the total scores of the raters is plotted. The solid thin line represents the zero axis. The bold black line represents the mean difference. The dotted lines indicate the limits of agreement. MD: Mean difference.

Visual inspection showed no systematic bias between sessions (Fig 1) or between raters (Fig 2), except for the TCMS total score between two of the three raters, where one rater scored systematically higher than the other rater (Fig 2A). This systematic bias was not present when these two raters were compared to the third rater (Fig 2B & 2C).

### Item-level reliability

Cohen’s kappa for test-retest reliability varied strongly from poor to good among single items of the TCMS, with only two items showing excellent reliability, namely items ‘5 left’ (κ = 1.00) and ‘11 b’ (κ = 1.00). Items with “*” could not be calculated due to a constant scoring on at least one of the testing moments. In contrast, percentage of agreement scores revealed good to excellent agreement, with only 5 items scoring below 70%. Interrater reliability for the items of the TCMS showed higher reliability scores, with most items ranging from moderate to excellent, and only three items having poor reliability, namely item ‘11a’ (κ = 0.33), ‘12a’ (κ = 0.33) and ‘12b’ (κ = 0.07). Percentage of agreement scores for interrater reliability showed similar results as the kappa scores. Exact values can be found in Table 3.

**Table 3:** Test-retest and interrater reliability of TCMS on item-level.

Subscale/Items	Test-retest (N = 10)			Interrater (N = 10)		
	Cohen’s κ	95% CI	%	Free marginal κ	95% CI	%
**Static sitting balance**						
1. Upright sitting	*		100%	1.00	1.00	100%
2. Lift both arms	*		100%	1.00	1.00	100%
3. Legs crossed by therapist (left)	*		100%	1.00	1.00	100%
3. Legs crossed by therapist (right)	*		100%	0.90	0.70–1.00	93%
4. Legs crossed by patient (left)	0.74	0.27–1.00	90%	0.64	0.36–0.93	80%
4. Legs crossed by patient (right)	0.62	-0.05–1.00	90%	0.73	0.47–1.00	80%
5. Leg abduction (left)	1.00	1.00	100%	0.73	0.47–1.00	80%
5. Leg abduction (right)	0.41	- 0.18–1.00	80%	0.64	0.36–0.93	73%
**Selective movement control**						
6a. Forward lean	*		100%	1.00	1.00	100%
6b. Compensations 6a	*		100%	0.87	0.61–1.00	93%
7a. Backward lean	*		100%	1.00	1.00	100%
7b. Compensations 7a	-0.25	-0.49 –-0.18	60%	0.60	0.20–1.00	80%
8a. Lateral lean (left)	*		100%	1.00	1.00	100%
8a. Lateral lean (right)	*		100%	1.00	1.00	100%
8b. Trunk strategy 8a (left)	0.62	-0.05–1.00	90%	0.60	0.20–1.00	80%
8b. Trunk strategy 8a (right)	*		100%	1.00	1.00	100%
8c. Compensations 8a (left)	0.60	0.15–1.00	80%	1.00	1.00	100%
8c. Compensations 8a (right)	0.58	0.07–1.00	80%	0.60	0.20–1.00	80%
9a. Pelvic lift (left)	*		100%	0.87	0.61–1.00	93%
9a. Pelvic lift (right)	*		100%	1.00	1.00	100%
9b. Trunk strategy 9a (left)	0.60	0.14–1.00	80%	0.60	0.28–0.92	73%
9b. Trunk strategy 9a (right)	0.35	-0.24–0.94	70%	0.80	0.54–1.00	87%
9c. Compensations 9a (left)	0.35	-0.24–0.94	70%	0.60	0.20–1.00	80%
9c. Compensations 9a (right)	0.55	0.04–1.00	80%	0.60	0.20–1.00	80%
10a. Upper trunk rotation	0.53	-0.05–1.00	80%	0.80	0.54–1.00	87%
10b. Head strategy 10a	0.62	-0.05–1.00	90%	1.00	1.00	100%
11a. Lower trunk rotation	0.80	0.44–1.00	90%	0.70	0.54–1.00	80%
11b. Pelvic compensation 11a	1.00	1.00	100%	0.33	-0.10–0.77	67%
12a. Forward and backward pelvic shuffle	0.78	0.39–1.00	90%	0.33	0.04–0.63	50%
12b. Compensations 12a	*		70%	0.07	-0.33–0.47	53%
**Dynamic reaching**						
13. Forward reaching	*		90%	1.00	1.00	100%
14. Lateral reach (left)	0.35	-0.24–0.94	70%	1.00	1.00	100%
14. Lateral reach (right)	*		90%	1.00	1.00	100%
15. Lateral cross-over reach (left)	0.78	0.38–1.00	90%	0.90	0.70–1.00	93%
15. Lateral cross-over reach (right)	0.07	-0.43–0.58	50%	0.70	0.40–1.00	80%

TCMS: Trunk Control Measurement Scale; Κ: Kappa; CI: Confidence interval; %: Percentage of agreement

*: calculation not possible

Similar results for test-retest reliability on item-level were found for BESTest. Kappa scores varied from poor disagreement to excellent agreement, with only four items having excellent agreement, namely item ‘2’ (κ = 0.83), ‘11 right’ (κ = 0.82), ‘19c’ (κ = 1.00) and ‘21’ (κ = 0.81). Items with “*” could not be calculated due to a constant scoring on at least one of the testing moments. Percentage of agreement scores revealed that only five items showed agreement of <70%. For interrater reliability, all items scored good to excellent. Exact values can be found in Table 4.

**Table 4:** Test-retest and interrater reliability of BESTest on item level.

Subscale/Items	Test-retest (N = 10)			Interrater (N = 10)		
	Cohen's κ	95% CI	%	Free marginal κ	95% CI	%
**Biomechanical constraints**						
1. Base of support	*		100%	0.91	0.74–1.00	93%
2. COM alignment	0.83	0.52–1;00	90%	0.73	0.47–1.00	80%
3. Ankle strength hand range	0.41	-0.26–1.00	80%	0.73	0.47–1.00	80%
4. Hip/trunk lateral strength	0.47	0.07–0.88	70%	0.82	0.59–1.00	87%
5. Sit on floor and stand up	*		100%	1.00	1.00	100%
**Stability limits/Verticality**						
6a. Lateral lean during sitting (left)	0.21	-0.43–0.85	70%	0.73	0.47–1.00	80%
6a. Lateral lean during sitting (right)	0.21	-0.43–0.85	70%	0.73	0.47–1.00	80%
6b. Verticality (left)	*		90%	0.82	0.59–1.00	87%
6b. Verticality (right)	*		90%	0.73	0.47–1.00	80%
7. Functional lean forward	-0.09	-0.67–0.50	50%	0.82	0.59–1.00	87%
8. Functional lean lateral (left)	0.62	0.17–1.00	80%	1.00	1.00	100%
8. Functional lean lateral (right)	0.52	-0.05–1.00	80%	1.00	1.00	100%
**Transistions-Anticipatory** **postural adjustments**						
9. Sit to stand	*		100%	1.00	1.00	100%
10. Rise to toes	0.58	0.10–1.00	80%	0.91	0.74–1.00	93%
11. Stand on one leg (left)	0.60	0.16–1.00	80%	0.64	0.36–0.93	73%
11. Stand on one leg (right)	0.82	0.51–1.00	90%	1.00	1.00	100%
12. Alternate stair touching	*		90%	1.00	1.00	100%
13. Standing arm raise	*		100%	0.91	0.74–1.00	93%
**Reactive postural responses**						
14. In place response–forward	0.57	0.06–1.00	80%	0.73	0.47–1.00	80%
15. In place response–backward	-0.06	-0.44–1.31	30%	0.91	0.74–1.00	93%
16. Compensatory stepping correction–forward	0.25	-0.35–0.85	70%	0.91	0.74–1.00	93%
17. Compensatory stepping correction–backward	0.49	-0.02–1.00	70%	0.82	0.59–1.00	87%
18. Compensatory stepping correction–lateral (left)	0.09	-0.47–0.66	60%	1.00	1.00	100%
18. Compensatory stepping correction–lateral (right)	0.17	-0.45–0.78	60%	0.82	0.59–1.00	87%
**Sensory orientation**						
19a. Sensory integration–eyes open, frim surface	*		100%	1.00	1.00	100%
19b. Sensory integration–eyes closed, frim surface	*		90%	1.00	1.00	100%
19c. Sensory integration–eyes open, foam surface	1.00	0.00–1.00	100%	0.91	0.74–1.00	93%
19b. Sensory integration–eyes closed, foam surface	-0.25	-0.50–0.00	40%	0.91	0.74–1.00	93%
20. Incline, eyes closed	0.40	-0.05–0.85	70%	0.73	0.47–1.00	80%
**Stability in gait**						
21. Walk on level surface	0.81	0.50–1.00	90%	1.00	1.00	100%
22. Change in gait speed	*		100%	1.00	1.00	100%
23. Walk with head turns	0.52	0.10–0.93	70%	0.91	0.74–1.00	93%
24. Walk with pivot turns	*		90%	1.00	1.00	100%
25. Step over obstacle	*		90%	0.91	0.74–1.00	93%
26. Timed “get up and go” test	*		100%	0.91	0.74–1.00	93%
27. Timed “Get up and go” test with dual task	0.51	0.08–0.95	70%	0.91	0.74–1.00	93%

BESTest: Balance Evaluation Systems Test; Κ: Kappa; CI: Confidence interval; %: Percentage of agreement

*: calculation not possible

### VAS scores

No significant differences in VAS scores were found between or within sessions. All scores can be found in Table 5.

**Table 5:** Visual analog scale scores for pain before, between and after measurement.

	VAS 1	VAS 2	VAS 3
Test session	1.65 ± 2.64	2.50 ± 3.02	2.55 ± 3.39
Retest session	1.95 ± 2.44	2.35 ± 2.70	2.00 ± 2.21
Interrater session	1.70 ± 2.10	1.90 ± 2.12	1.80 ± 2.08

VAS 1: before TCMS; VAS 2: between TCMS and BESTest; VAS 3: after BESTest

## Discussion

Recent research highlighted the potential of dynamic parameters, such as balance control, in the evaluation of ASD patients [4,5,24]. These findings stress the need for reliable and valid tools to measure balance in this population. Therefore, the purpose of this study was to assess reliability of two existing balance scales in the ASD population.

Good to excellent test-retest and interrater reliability was found for both TCMS and BESTest, based on high ICC’s, small 95% CI’s and relatively small SEM’s on the total scores. Therefore, it can be concluded that these tests are reliable to assess balance for this population. For the BESTest, the excellent ICC score for interrater reliability (0.94) was similar to that of Horak et al. (0.91) [15]. Wang-Hsu et al. found an excellent ICC (0.93) for test-retest reliability, similar to the result of this study (0.90) [25]. Both studies investigated balance in elderly, with and without balance disorders, but since the current study is the first to test reliability of TCMS and BESTest in the ASD population, our results could not be compared with other studies in this specific population. For the TCMS total score, good ICC scores for both test-retest (0.88) and interrater (0.76) reliability were found. However, these ICC scores were lower than those found in a CP population, with Heyrman et al. [16] and Marsico et al. [26] reporting scores above 0.90. Since the TCMS was originally designed for the CP population, this difference in population might explain the differences in reliability scores.

Also the reliability of the subscales was investigated. Although the interrater ICC scores of almost all subscales were good (>0.75)—except for ‘static sitting balance’ of TCMS and ‘sensory orientation’ of BESTest—all were lower than those of the total scores. This difference even increased for test-retest reliability with poor ICC’s for ‘stability limits/verticality’ and ‘postural reactive responses’ of BESTest and ‘dynamic reaching’ of TCMS. Also the increased 95% CI’s, with some even becoming negative between sessions, indicating a relation of disagreement, as well as the increased SEM’s indicated a decreased reliability on subscale-level. These results are not in line with the results found in other reliability studies on BESTest and TCMS, which reported good to excellent reliability on subscale-level for all subscales, both between sessions and raters [15,16,25]. A possible explanation might be found in the spinal deformity of these subjects and their musculoskeletal compensation strategies. To keep the trunk as upright as possible and the COM within the BOS, these patients are constantly compensating [6–11]. Literature suggests that fatigue might impact this maintenance of an upright posture after ten minutes walking [27]. Accordingly, fatigue during the balance assessment might have induced variations in the testing position within the subject. This assumption might explain why especially reaching tasks have lower reliability, since the zero position in these tasks completely depends on the subject-specific upright position the patient is able to adopt at that point in time. Since ASD patients compensate during normal standing, it is not surprising they have difficulties during out-of-plane movements, such as in the ‘reactive postural response’ subscale of the BESTest, which might influence their day-to-day performance. These assumptions were also confirmed by the fact that reliability between sessions, containing repeated measurements, of most subscales was lower than between raters. This suggests that decreased reliability is mainly due to differences in instructions and/or performance, rather than differences in interpretation by the raters. Potter et al. investigated the test-retest reliability of the BESTest in multiple sclerosis and also found lower ICC’s for ‘stability limits/verticality’ and ‘reactive postural response’ [28], however still higher than the ICC’s of the current study. The difference between test-retest and interrater reliability of ‘dynamic reaching’ of the TCMS was also seen by Heyrman et al. [16], confirming our findings. This decreased reliability on subscale-level makes it difficult to reliably attribute a balance deficit to an impairment on a specific balance subsystem represented by the respective subscales.

To identify those items who contribute most to the decreased reliability on subscale-level, reliability analysis on item-level was performed. Lower test-retest reliability compared to interrater reliability was also clearly present on item-level. Not surprisingly, the items of the least reliable subscales (‘dynamic reaching’ of TCMS and ‘stability limits/verticality’ and ‘reactive postural response’ of BESTest) also contained the most unreliable items. Possible reasons for this low test-retest reliability were already described above. Between raters, items of both TCMS and BESTest were clearly more reliable. Besides of differences in day-to-day performance, also statistical methods might have influenced the results. Firstly, Cohen’s kappa is, in contrast to the free marginal kappa, a very strict statistic, which might not be very appropriate when there is a large proportion of agreement. Also, if this agreement goes along with a small amount of variability in ratings among subjects, such as a maximum score for all subjects by all raters, Cohen’s kappa can often not be calculated [29]. In contrast, perfect agreement in combination with no variability in ratings, results in a free marginal kappa of 1 [21]. Therefore, percentages of agreement are useful to check if the lack of a high kappa score was either due to a high amount of disagreement or rather due to a lack of variability in the ratings between subjects. This lack of variability in ratings between subjects, might point out that many items of both TCMS and BESTest are subject to a ceiling effect, questioning the use of these items in the ASD population. BESTest and TCMS already showed to be able to discriminate ASD patients from healthy adults on balance performance [4]. However, to be used in a clinical setting, the ability to discriminate between subgroups within the ASD population, e.g. different deformity types, or as a follow-up tool pre- and post-intervention, is even more important. A good reliability on scale-level and decreased reliability on subscale- and item-level suggests that BESTest and TCMS are valid tools to discriminate between ASD patients with and without balance problems. Yet, an ASD specific balance scale might be needed for further discrimination between subgroups or within subjects between pre- and post-intervention. The results of the current study, in terms of reliability and ceiling effects, might help to select those items which show the highest discriminative potential. However, to reach the goal of developing an ASD specific balance scale, a more extended data collection is necessary.

### Limitations

The sample size of this study is relatively small. This might have been a reason for the lack of variability in ratings between subjects. However, other studies investigating the reliability of BESTest and TCMS [16,28] show very similar reliability scores, which indirectly indicates the sample size in this study to have been adequate. As previously mentioned differences in day-to-day performance can influence the results. To control for the influence of pain, VAS scores were obtained. No significant differences were found between the test and retest sessions. At last, since both the test and the retest were administered by the same rater, one could argue these results rather represent a mixture of both intrarater and test-retest reliability [20]. However, since a balance test is subject to day-to-day performances of the participants, especially in patients, and testing moments were not on the same day, test-retest reliability will have been the main contributing factor of the two. The study did not contain a control group, making it difficult to compare our results to the literature. Therefore, although all raters were trained before data collection, differences in rating might have influenced the results.

## Conclusion

To our knowledge this is the first study evaluating the reliability of balance scales in ASD. Based on the reliability analysis from scale- to item-level, it can be concluded that both TCMS and BESTest are reliable tools to assess balance in ASD patients. However, looking deeper into the reliability of subscales and single items revealed lower levels of reliability compared to the total scores. Based on these results, the question is raised whether there is need for an ASD-specific balance scale, containing reliable items and subscales, which specifically aims at the balance impairments of ASD patients. Future research should focus on the development of such a pathology-specific scale, which is able to discriminate, not only between ASD patients and healthy controls, but even more importantly between subgroups within the ASD population or within subjects as a follow-up tool pre- and post-intervention.
